# Supplementary material for: Contactin-1 is a critical neuronal cell surface receptor for perineuronal net structure
Source: J Biol Chem. 2025 Apr 10;301(5):108504. doi: 10.1016/j.jbc.2025.108504 (PMC12139414; doi:10.1016/j.jbc.2025.108504)
Supplement: Supplemental Figure 1 [file mmc1.pdf]

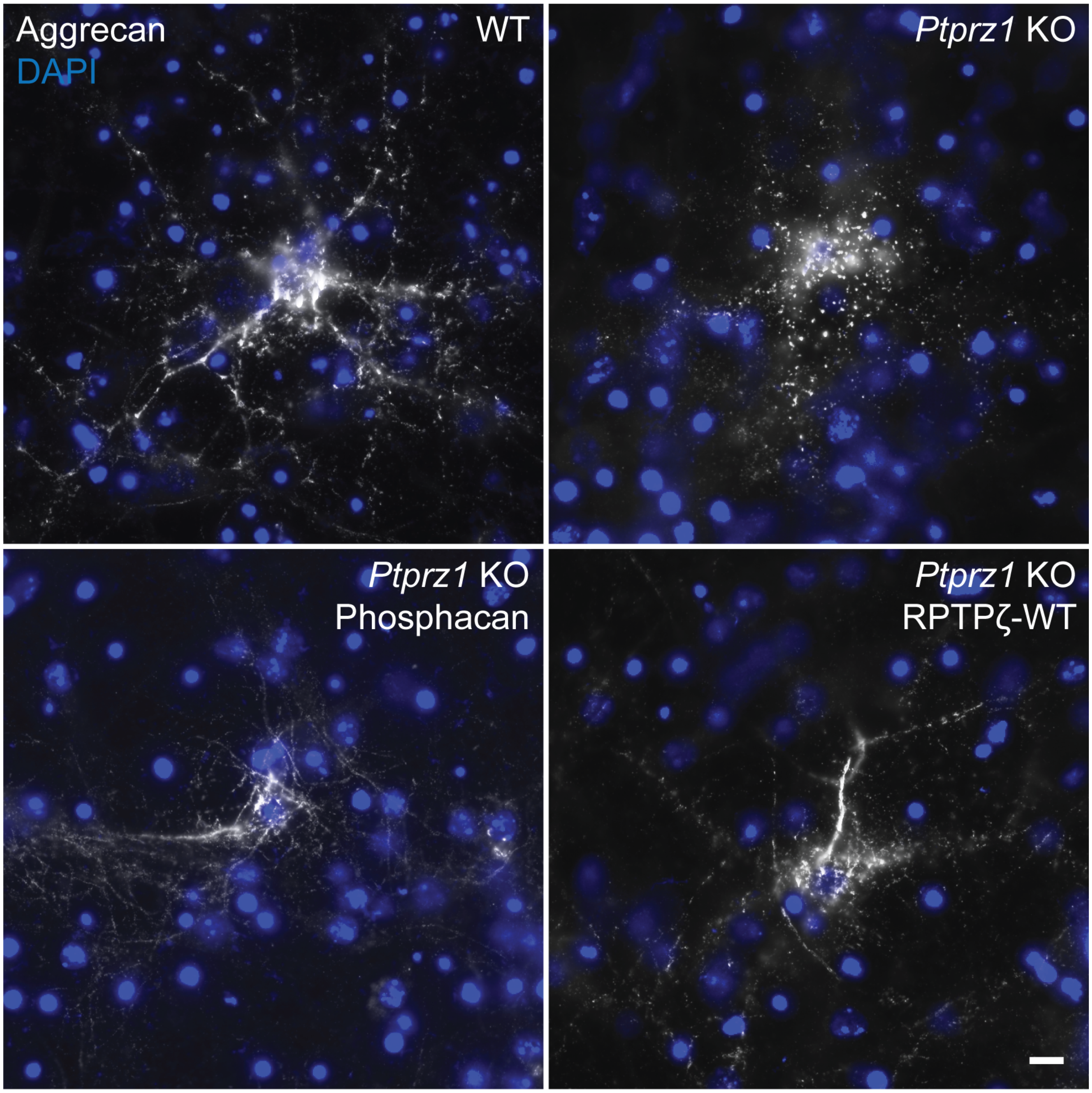

Figure S1. Disrupted PNN Structure in *Ptprz1* KO cultures can be recovered with purified phosphacan or recombinant RPTPζ. Neurons derived from E16 WT mice were positive for PNN component aggrecan and displayed regular and continuous staining. Aggrecan staining in cultures from *Ptprz1* KO mice was disrupted and appeared broken, aggregated, and punctate. Addition of phosphacan purified from WT brain homogenates to *Ptprz1* KO cells restored PNN component binding and structure. As above, addition of recombinant RPTPζ (RPTPζ-WT) to *Ptprz1* KO cells restored PNN component binding and structure. These findings are consistent with previously published studies(22, 24). Scale bar, 10 μm.
